# *In Vitro* Antitumor Activity of Stellettin B, a Triterpene from Marine Sponge *Jaspis stellifera*, on Human Glioblastoma Cancer SF295 Cells

**DOI:** 10.3390/md12074200

**Published:** 2014-07-15

**Authors:** Sheng-An Tang, Qianxiang Zhou, Wen-Zhi Guo, Yuling Qiu, Ran Wang, Meihua Jin, Wenjing Zhang, Ke Li, Takao Yamori, Shingo Dan, Dexin Kong

**Affiliations:** 1 Tianjin Key Laboratory on Technologies Enabling Development of Clinical Therapeutics and Diagnostics, School of Pharmaceutical Sciences and Research Center of Basic Medical Sciences, Tianjin Medical University, Tianjin 300070, China; E-Mails: tangshengan@tijmu.edu.cn (S.-A.T.); zqx9260@163.com (Q.Z.); qiuyuling@tijmu.edu.cn (Y.Q.); wangran@tijmu.edu.cn (R.W.); jinmeihua@tijmu.edu.cn (M.J.); 2 Division of Molecular Pharmacology, Cancer Chemotherapy Center, Japanese Foundation for Cancer Research, 3-8-31, Ariake, Koto-ku, Tokyo 135-8550, Japan; E-Mails: guo.wenzhi@jfcr.or.jp (W.-Z.G.); yamorit@jfcr.or.jp (T.Y.); 3 School of Chemistry, Shandong University, 27 Shanda South RD, Jinan 250100, China; E-Mail: 68875299@163.com; 4 Department of Obstetrics and Gynecology, Tianjin University Hospital, Tianjin 300072, China; E-Mail: likeleaf77@gmail.com

**Keywords:** stellettin B, antitumor activity, apoptosis, p-Akt, *in vitro*

## Abstract

Stellettin B was isolated from marine sponge *Jaspis stellifera*. *In vitro* antitumor activities were investigated on 39 human cancer cell lines. Stellettin B exhibited highly potent inhibition against the growth of a human glioblastoma cell line SF295, with a GI50 of 0.01 μM. In contrast, stellettin B showed very weak inhibitory activity on normal cell lines including HMEC, RPTEC, NHBE and PrEC, with GI50s higher than 10 μM, suggesting its relatively selective cytotoxicity against human cancer cells compared to normal human cell lines. We then focused on the antitumor activity of this compound on SF295 cells. Flow cytometric analysis indicated that stellettin B induced apoptosis in SF295 cells in a concentration-dependent manner. Further study indicated that stellettin B increased the production of ROS, the activity of caspase 3/7, as well as the cleavage of PARP, each of which is known to be involved in apoptosis. To investigate the molecular mechanism for cell proliferation inhibition and apoptosis induction, effect on the phosphorylation of several signal proteins of PI3K/Akt and RAS/MAPK pathways was examined. Stellettin B inhibited the phosphorylation of Akt potently, with no activity on p-ERK and p-p38, suggesting that inhibition of PI3K/Akt pathway might be involved in the antiproliferative and apoptosis-inducing effect. However, homogenous time-resolved fluorescence (HTRF) assay indicated that stellettin B did not inhibit PI3K activity, suggesting that the direct target might be signal protein upstream of Akt pathway other than PI3K.

## 1. Introduction

As a part of our discovery of new anticancer drug candidates from natural resources, we have been trying to search molecular-targeted antitumor lead compounds from marine organisms [1,2,3]. Until now, we have found several interesting compounds including cancer cell differentiation inducers, cell cycle arrest inducers from marine sponges [1,2,3].

Glioblastoma, the most common intracranial malignancy, constitutes about half of all gliomas. Due to its location in the brain, invasive behavior and poor prognosis, glioblastoma has become one of the most devastating cancers [4]. The overall median survival time is about 15 months despite the combined therapy of surgery, radiation and chemotherapy. Therefore, a more effective antitumor drug candidate for glioblastoma therapy has been expected [4].

**Figure 1:**
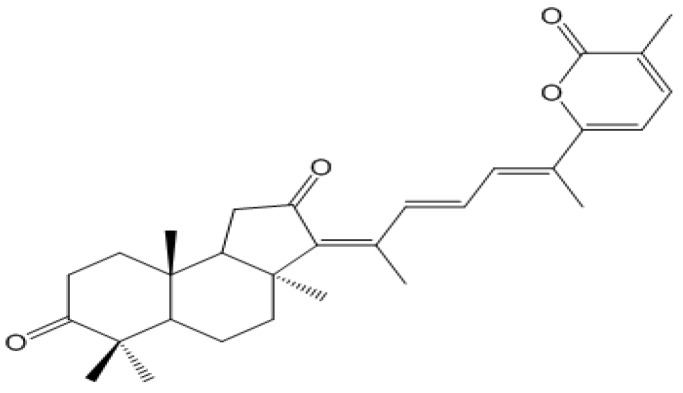
Chemical structure of stellettin B.

We recently isolated stellettin B (Figure 1) from marine sponge *Jaspis stellifera*, and evaluated the *in vitro* antitumor activity by use of a panel of 39 human cancer cell lines. Interestingly, stellettin B showed highly potent activity on human glioblastoma cancer SF295 cells. In contrast, this compound indicated very weak inhibition against several normal cell lines, suggesting its relatively selective cytotoxicity against human cancer cells compared to normal human cell lines. Therefore, we further examined the antitumor effect of stellettin B on SF295 cells *in vitro* and the underlying molecular mechanism.

## 2. Results and Discussion

### 2.1. Stellettin B Inhibited Cell Growth of Various Tumor Cell Lines Including SF295

**Figure 2:**
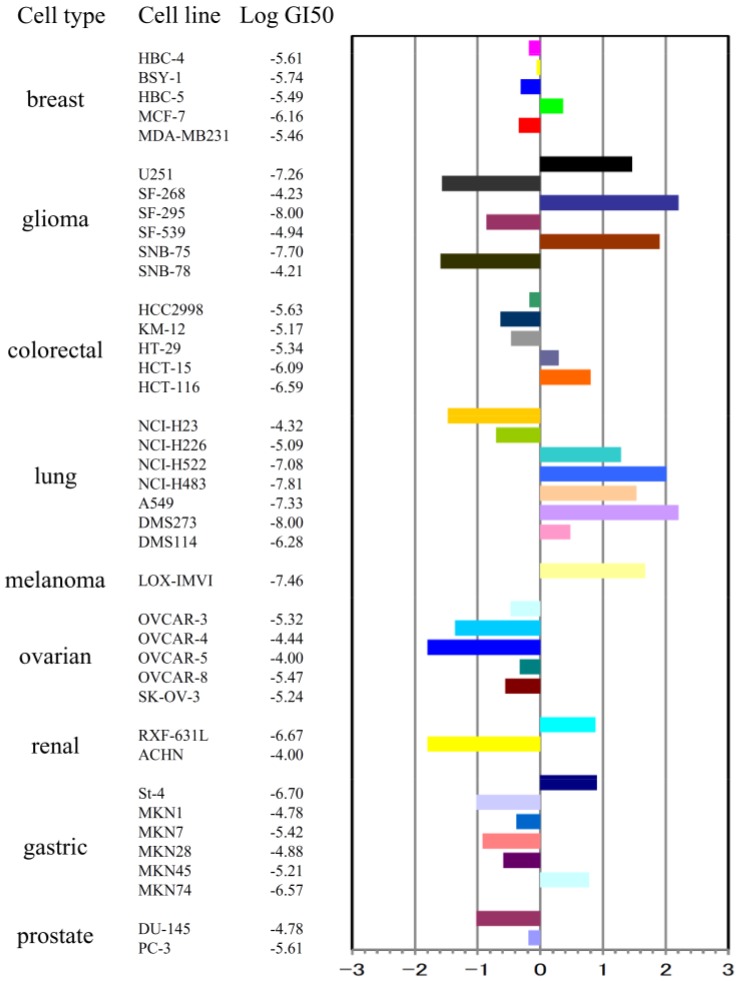
Effect of stellettin B on cell growth of 39 human cancer cell lines. The Log GI50 values of stellettin B for the cell lines in JFCR39 panel, and the JFCR39 fingerprint which is plotted based on the Log GI50 values [5], are indicated. In the fingerprint, The X-axis shows difference in logarithmic scale between the mean of Log GI50 values for all 39 cell lines (expressed as 0 in the fingerprint) and the Log GI50 for each cell line in JFCR39 panel. **Columns to the right of 0** indicate the sensitivity of the cell lines to stellettin B and **columns to the left** indicate the resistance.

To investigate the *in vitro* antitumor activity of stellettin B, we first determined the inhibitory effect on the cell growth of 39 human cancer cell lines (JFCR39) by use of sulforhodamine B (SRB) assay, as described by us previously [5,6]. The GI50 value (the concentration of a given compound required for 50% growth inhibition of cells) for each cancer cell line was obtained, and the JFCR39 fingerprint was plotted based on the Log GI50 values (Figure 2).

Among the 39 cell lines, human glioblastoma cell SF295 exhibited high sensitivity to stellettin B, with the Log GI_50_ as −8.00 (GI_50_ as 0.01 μM), displaying potent antitumor activity of stellettin B on SF295 cells.

### 2.2. Stellettin B Showed High Selectivity in Growth Inhibition against SF295 Cells Compared with Normal Cells

**Figure 3:**
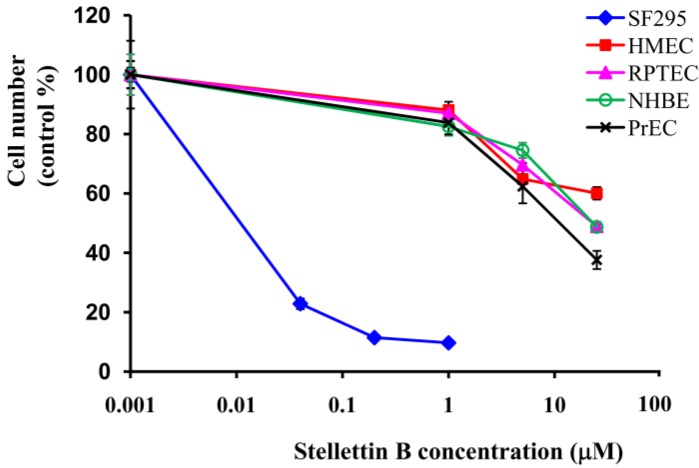
Inhibitory effect of stellettin B on cell growth of normal cell human mammary epithelial cells (HMEC), renal proximal tubule epithelial cells (RPTEC), normal human bronchial epithelial cells (NHBE), human prostate epithelial cells (PrEC), as well as cancer cell SF295. After treatment with various concentrations of stellettin B, cell number was determined by WST assay, and expressed as the percentage of control (cells without stellettin B treatment).

We then investigated the inhibition of stellettin B against growth of normal cells. Several normal cell lines including normal human mammary epithelial cells (HMEC), human renal tubule epithelial cells (RPTEC), normal human bronchial epithelial cells (NHBE), normal human prostate epithelial cells (PrEC) were used. Cell viability was determined by use of WST assay after treatment with various concentrations of stellettin B for 48 h. Interestingly, in contrast to the potent inhibition against SF295 cells (GI_50_ = 0.03 μM), very weak activity (GI_50_ > 10 μM) was shown on each of the four normal cell lines, indicating that SF295 cells are significantly more sensitive to stelletin B than the normal cell lines tested (Figure 3).

### 2.3. Stellettin B Induced Apoptosis in SF295 Cells

**Figure 4:**
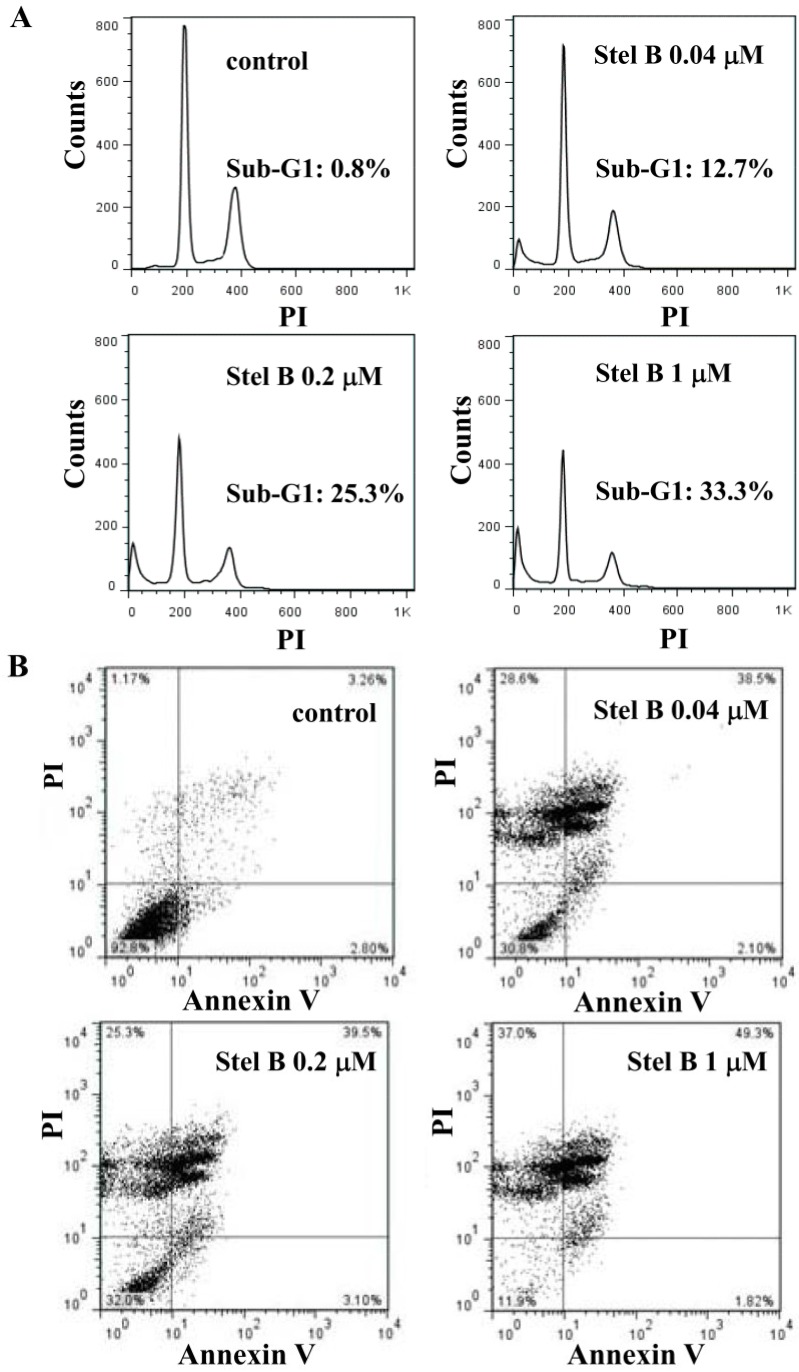
Stellettin B (Stel B) induced apoptosis in SF295 cells. SF295 cells were incubated with various concentrations of stellettin B for 24 h. (**A**) The collected cells were dyed with propidium iodide (PI) and analyzed by flow cytometer; (**B**) The collected cells were stained with Annexin V/PI, and analyzed by flow cytometer.

We then investigated the effect of stellettin B on the cell cycle progression and apoptosis in SF295 cells by flowcytometric analysis. The cells were treated with 0, 0.04, 0.2, and 1 μM of stellettin B for 24 h and the DNA content was measured by propidium iodide staining method using flow cytometer. As shown in Figure 4A, while no apparent cell cycle arrest was observed, the sub-G1 population (apoptotic cells) increased concentration-dependently after treatment by stellettin B, with the percentages to be 0.8%, 12.7%, 25.3% and 33.3%, respectively, suggesting that stellettin B treatment induced apoptosis in SF295 cells.

We further investigated the apoptosis as well as the stage of that induced in SF295 cells by stellettin B. The cells were stained by using Annexin V/PI double staining after treatment with 0, 0.04, 0.2, and 1 μM of stellettin B for 24 h, and analyzed by flow cytometer. As shown in Figure 4B, stellettin B obviously increased the population of SF295 cells in the upper-right quadrant (both Annexin V and PI positive), indicating that stellettin B induced the late-stage apoptosis [7] in SF295 cells concentration-dependently.

### 2.4. Stellettin B Increased Caspase 3/7 Activity and the Cleavage of Poly-(ADP-Ribose) Polymerase (PARP) in SF295 Cells

**Figure 5:**
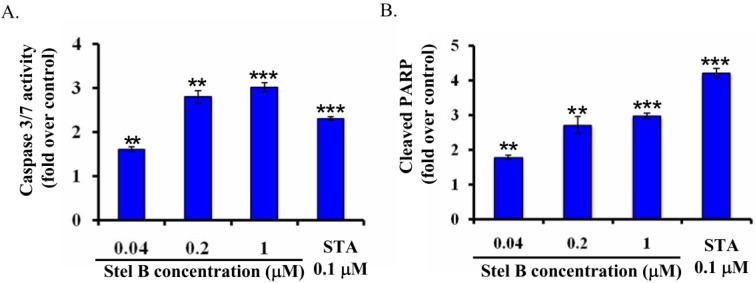
Stellettin B (Stel B) increased caspase 3/7 activity and the cleavage of PARP in SF295 cells. (**A**) Caspase 3/7 activity in SF295 cells after treatment with stellettin B was determined by Caspase-Glo 3/7 Assay, and expressed as the fold over that in control (cells without treatment). Staurosporine (STA) was used as a positive control. ** *p* < 0.01; *** *p* < 0.001, compared with control. Data represent three independent experiments, each of which was carried out in triplicate; (**B**) The amount of cleaved PARP in SF295 cells after treatment with stellettin B was determined by PathScan ELISA, and shown as the fold over that in control (cells without treatment). STA was used as a positive control. ** *p* < 0.01; *** *p* < 0.001, compared with control. Data represent two independent experiments, each of which was carried out in triplicate.

To confirm the apoptosis-inducing activity of stellettin B and investigate the related mechanism on SF295 cells, we determined caspase 3/7 activity in SF295 cells with or without stellettin B treatment by using Caspase-Glo assay [8]. As shown in Figure 5A, caspase 3/7 activity increased concentration-dependently in stellettin B treated SF295 cells, supporting that apoptosis was induced by stellettin B. Staurosporine (STA), which is known as an apoptosis-inducing agent [9] and therefore used as positive control, also increased caspase 3/7 activity significantly at 0.1 μM.

PARP is a key signaling nuclear protein involved in DNA repair and apoptosis. As a downstream substrate, PARP is cleaved by activation of caspase-3. Therefore, cleavage of PARP is also widely used as an indicator of apoptosis [10]. To further demonstrate the apoptosis-inducing activity of stellettin B on SF295 cells, we then determined the cleavage of PARP with PathScan ELISA assay. As shown in Figure 5B, the cleaved PARP increased concentration-dependently after treatment with stellettin B for 24 h, suggesting that apoptosis in SF295 cells might be induced through cleavage of PARP by activation of caspases.

### 2.5. Stellettin B Increased the Reactive Oxygen Species (ROS) in SF295 Cells

**Figure 6:**
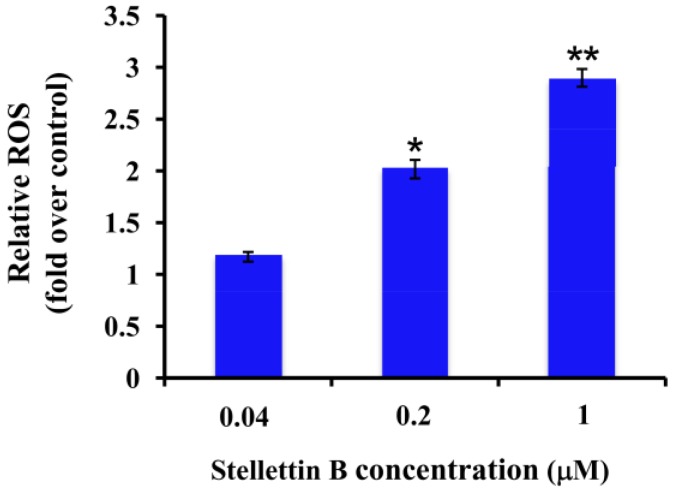
Stellettin B increased ROS production in SF295 cells. SF295 cells were incubated with various concentrations of stellettin B for 24 h. Then the cells were stained with CM-H_2_DCFDA, and the fluorescent intensity was determined by excitation at 485 nm and emission at 535 nm using a multi-mode microplate reader. The amount of ROS in stellettin B treated cells is shown as the fold over that in cells without treatment (control). * *p* < 0.05; ** *p* < 0.01, compared with control. Data represent two independent experiments, each of which was carried out in triplicate.

Reactive oxygen species (ROS) are known to play an important role in apoptosis [11]. Excess ROS induce oxidative modification of cellular macromolecules, activate caspases and promote apoptotic cell death [11]. Since our result above showed that stellettin B induced apoptosis and activated caspase 3/7, we then investigated whether stellettin B increased ROS or not. As indicated in Figure 6, the amount of ROS increased in a concentration-dependent manner after treatment with stellettin B, suggesting that the ROS production is promoted by stellettin B.

### 2.6. Stellettin B Inhibited Phosphorylation of Akt in SF295 Cells

**Figure 7:**
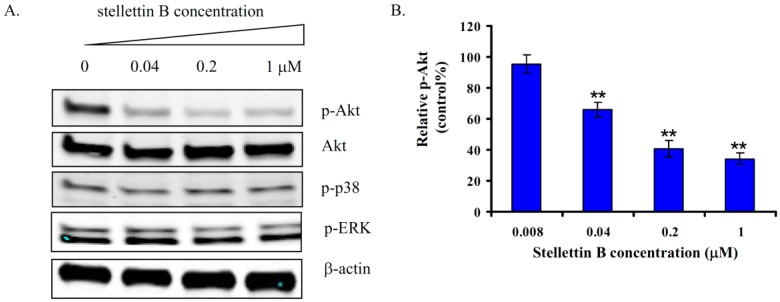
Effect of stellettin B on the phosphorylation of Akt, p38 and ERK in SF295 cells. (**A**) SF295 cells were incubated with 0, 0.04, 0.2, 1 μM of stellettin B for 24 h. Cell lysates were prepared and applied to 4%–20% SDS-PAGE. After being transferred to Immobilon-FL PVDF membrane, the blots were exposed to anti-p-Akt, anti-Akt, anti-p-p38, anti-p-ERK or anti-β-actin, and then to Alexa Fluor 680 anti-rabbit IgG secondary antibodies. Signals from the bound labeled-antibodies were detected using the Odyssey Infrared Imaging System; (**B**) SF295 cells were incubated with various concentrations of stellettin B for 24 h. The amount of p-Akt (Ser473) after treatment was determined by PathScan ELISA, and shown as the percentage of that in cells without treatment (control). ** *p* < 0.01, compared with control. Data represent two independent experiments, each of which was carried out in triplicate.

To investigate the mechanism for stellettin B to induce apoptosis and inhibit growth of SF295 cells, we examined the effects on several signal proteins of main pathways which are involved in cell survival and growth, including Akt, p38 and ERK. As shown in Figure 7A, treatment with stellettin B for 24 h inhibited phosphorylation of Akt concentration-dependently, while no obvious change was observed in the phosphorylation of p38 and ERK.

To confirm the inhibition of stellettin B against the phosphorylation of Akt, we further determined the amount of phosphorylated Akt in stellettin B-treated SF295 cells by use of PathScan ELISA assay. Figure 7B indicated the relative amount of p-Akt in SF295 cells after treatment with 0.008, 0.04, 0.2, and 1 μM of stellettin B. The phosphorylation of Akt was indeed inhibited in a concentration-dependent manner by stellettin B.

**Figure 8:**
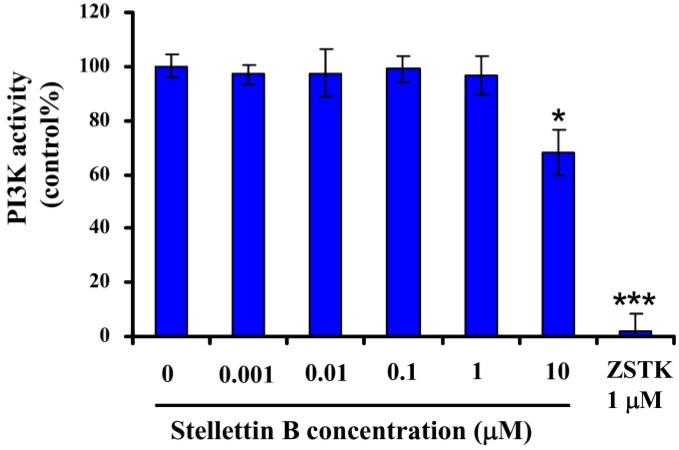
Effect of stellettin B on the activity of recombinant PI3K. The recombinant PI3Kα was incubated with various concentrations of stellettin B, in presence of 10 μM PIP2 and 10 μM ATP for 30 min in the wells of a 384-well plate at room temperature. After the addition of the stop solution (containing EDTA and biotin-PIP3) and the detection solution, the resulting mixture was further incubated for 14 h. Signals were read using a multi-mode microplate reader. The PI3K activity of a certain sample was calculated as described in the Experimental Section. Representative data from two independent experiments, each carried out in triplicate, were used for plotting. * *p* < 0.05; *** *p* < 0.001, compared with control.

PI3K/Akt pathway plays a key role in cell survival, growth, *etc.* [12,13]. Blockage of this pathway has been known as a promising approach for cancer therapy [14,15]. Our result showed that stellettin B inhibited phosphorylation of Akt, which might be involved in the antiproliferative and apoptosis-inducing effect of the compound on SF295 cells. Since inhibition against phosphorylation of Akt might be attributed to inactivation of the upstream signal proteins like PI3K, and Receptor tyrosine kinases (RTKs), *etc.* we then first determined the activity of stellettin B on PI3K by use of Homogenous time-resolved fluorescence (HTRF) assay [16]. As shown in Figure 8, in contrast to the potent activity of ZSTK474 which is a PI3K inhibitor [16], stellettin B indicated no inhibition against PI3K activity at concentrations up to 1 μM which is 100 fold of its GI50 for SF295 cells, suggesting that the antiproliferative and apoptosis-inducing activity is not attributed to PI3K inhibition. Therefore, the direct target of stellettin B might be signal protein upstream of Akt other than PI3K. Identification of the direct target of stellettin B is ongoing.

Stellettin B was previously reported to exhibit cytotoxic activity on human promyelocytic leukemia HL60 cells [17], and human colon cancer cell HCT-116 [18]. Moreover, the analogues were reported to induce apoptosis and G1 arrest in hepatoma tumor cells [19,20]. However, to our knowledge, there is no report about the selectivity of the compound against cancer cells compared to normal cells. Moreover, there is no report about the mechanism for the antiproliferative and apoptosis-inducing effect of stellettin B until now. Our study showed that stellettin B induced apoptosis by targeting other signal protein upstream of Akt than PI3K.

## 3. Experimental Section

### 3.1. Materials

Caspase-Glo 3/7 Assay Kit was obtained from Promega Corporation (Madison, WI, USA). FITC Annexin V Apoptosis Detection kit was from BD Pharmingen (Bedford, MA, USA). Anti-p-Akt (ser473), anti-Akt, anti-p-p38, anti-p-ERK antibodies, PathScan cleaved PARP and PathScan Phospho-Akt1 Sandwich ELISA Kits were from Cell Signaling Technology Inc. (Danvers, MA, USA). Anti-β-actin and propidium iodide were from Sigma (St. Louis, MO, USA). The recombinant PI3Kα and the PI3K HTRF Assay Kit were purchased from Millipore (Billerica, MA, USA).

### 3.2. Cell Lines and Cell Culture

A panel of 39 human cancer cell lines [21,22], which consists of the following cell lines: lung cancer, NCI-H23, NCI-H226, NCI-H522, NCI-H460, A549, DMS273 and DMS114; colorectal cancer, HCC-2998, KM-12, HT-29, HCT-15 and HCT-116; gastric cancer, MKN-1, MKN-7, MKN-28, MKN-45, MKN-74 and St-4; ovarian cancer, OVCAR-3, OVCAR-4, OVCAR-5, OVCAR-8 and SK-OV-3; breast cancer, BSY-1, HBC-4, HBC-5, MDA-MB-231 and MCF-7; renal cancer, RXF-631L and ACHN; melanoma, LOX-IMVI; glioma, U251, SF-295, SF-539, SF-268, SNB-75 and SNB-78; prostate cancer, DU-145 and PC-3, was cultured in RPMI 1640 medium supplemented with 5% fetal bovine serum and kanamycin (100 U/mL) at 37 °C in a humidified atmosphere containing 5% CO_2_. Normal human mammary epithelial cells (HMEC), human renal proximal tubule epithelial cells (RPTEC), normal human bronchial epithelial cells (NHBE), normal human prostate epithelial cells (PrEC) were cultured in mammary epithelial growth medium (MEGM Bullet Kit, Lonza Corporation, Basel, Switzerland), renal epithelial growth medium (REGM Bullet Kit, Lonza Corporation, Basel, Switzerland), bronchial epithelial growth medium (BEGM Bullet Kit, Lonza Corporation, Basel, Switzerland), and prostate epithelial growth medium (PrEGM Bullet Kit, Lonza Corporation, Basel, Switzerland), respectively at 37 °C in a humidified atmosphere containing 5% CO_2_.

### 3.3. Isolation and Identification of Stellettin B

Stellettin B was isolated from the marine sponge *Jaspis·stellifera*, which was collected from the South China Sea. The frozen sponge was homogenized and soaked with methanol overnight. The resulting methanol extract was evaporated, and the residue was subjected to solvent partition between H_2_O and CH_2_Cl_2_ to give portions soluble in CH_2_Cl_2_. The CH_2_Cl_2_ portion was then separated by repeated SiO_2_ column and high-performance liquid chromatography to obtain stellettin B (248.0 mg from 1.5 kg sponge). The compound was identified by comparison of the mass and NMR data with those reported previously [23].

### 3.4. Determination of Inhibitory Activity on Cell Growth of 39 Human Cancer Cell Lines and Plotting of JFCR39 Fingerprint

Growth inhibition of cancer cells was assessed by the change in total cellular protein following 48 h treatment with stellettin B, and was measured by sulforhodamine B (SRB) assay as described by us previously [6]. The concentration of stellettin B required for 50% growth inhibition (GI50) of cells was calculated. The graphic representation (termed fingerprint) of the mean differential growth inhibition for the cells used in the JFCR39 panel was plotted based on a calculation that uses a set of Log GI50 values [6].

### 3.5. Determination of Inhibitory Activity on Growth of Normal Cells as well as SF295 Cancer Cells by WST Assay

WST assay was used to evaluate the inhibitory effect on growth of normal cells as well as cancer cell SF295, as described previously by us [24]. Briefly, 0.1 mL of cell suspensions was incubated in the respective media in 96-well plate at 37 °C in a humidified atmosphere containing 5% CO_2_. After treatment with various concentrations of stellettin B for 48 h, 10 μL of WST-8 was added to each well. Three hours later, the absorbance at 450 nm was measured by microplate spectrophotometer (BIO-RAD iMark, Hercules, CA, USA). The number of viable cells after treatment was calculated using the following formula: Cell number (% control) = 100 × (absorbance of a given sample − absorbance of Blank well)/(absorbance of Control well − absorbance of Blank well), where the Blank well contained medium but no cells and the Control well contained cells but no stellettin B. Three independent experiments were carried out.

### 3.6. Flow Cytometric Analysis of Cell Cycle Distribution and Apoptosis

The suspension (2 × 10^5^ cells/2 mL/well) of SF295 cells was placed in a 6-well plate and incubated for 24 h at 37 °C under a 5% CO_2_ atmosphere. Various concentrations of stellettin B (0, 0.04, 0.2, 1 μM) were added and further incubated for 24 h. Then the cells were harvested and washed twice with cold PBS, and fixed in 80% ethanol. The fixed cells were further washed with PBS, resuspended in 10 μg/mL ribonuclease A, and incubated at 37 °C for 30 min, The cells were dyed with PI (propidium iodide) solution (50 μg/mL) for 15 min at room temperature in the dark to be available for cell cycle analysis. The analysis was carried out by flow cytometer (FACS Calibur, Beckton Dickinson, Franklin Lakes, NJ, USA) provided with the CellQuest software (Braintree, MA, USA).

### 3.7. Annexin V/PI Assay for Apoptosis

Annexin V and PI staining assay was conducted to detect apoptosis as well as the stage of that induced by stellettin B, as described by us previously [8]. Briefly, SF295 cells grown in 12-well plates after treatment with various concentrations of stellettin B were collected, washed with PBS twice, stained with 5 μL of Annexin V-FITC and 5 μL of PI (5 μg/mL) in binding buffer for 15 min at room temperature in the dark, and analyzed by flow cytometer (FACS Calibur, Beckton Dickinson, Franklin Lakes, NJ, USA).

### 3.8. Caspase-Glo Assay

Caspase-Glo 3/7 assay was carried out as described by us previously [8] but with a small modification. One hundred μL of SF295 cell suspension was planted in the wells of a white 96-well plate. After incubation at 37 °C for 24 h, 50 μL of media with various concentrations of stellettin B was added. Twenty four hours later, an equal volume of Caspase-Glo 3/7 assay buffer was added and incubated at room temperature for 30 min. Then, the produced luminescence was measured using the multi-mode microplate reader (FilterMax F5, Molecular Devices, Sunnyvale, CA, USA). The caspase 3/7 activity of cells treated with stellettin B was expressed as the fold of the luminescence intensity produced over that produced by the control cells (treated with DMSO). Representative data from two independent experiments, each carried out in triplicate, were used. Student’s *t*-test was conducted for statistical analysis.

### 3.9. PathScan ELISA Assay

Pre-confluent cancer cells were treated with various concentrations of stellettin B in 6 cm dish. Three hours later, media were aspirated and the cells were washed with cold PBS. Then 0.4 mL of cold lysis buffer (20 mM Tris-HCl, 150 mM NaCl, 1 mM EDTA, 1 mM EGTA, 1% Triton, 2.5 mM sodium pyrophosphate, 1 mM β-glycerophosphate, 1 mM Na_3_VO_4_ and 1 μg/mL leupeptin) was added and kept on ice for 5 min. After being scraped off, the cells were collected, sonicated and centrifuged to make the resulting supernatant (cell lysate) available for PathScan ELISA assay. The assay was carried out according to the manufacturer’s instructions. Briefly, one hundred μL of cell lysate was added to each well of the anti-cleaved PARP or anti-p-Akt (Ser473) coated microwell plate, and then incubated for 2 h at 37 °C. After being washed, the bound protein was incubated with detection antibody, followed by exposure to the HRP (horseradish peroxidase)-linked secondary antibody and the TMB (tetramethyl benzidine) substrate. Finally, stop solution was added to each well and the absorbance at 450 nm was read by microplate spectrophotometer (BIO-RAD iMark). Student’s *t*-test was conducted for statistical analysis.

### 3.10. ROS Production Assay

ROS production was determined by staining the cells with CM-H_2_DCFDA (Thermo Fisher, Waltham, MA, USA) [8]. After treatment with various concentrations of stellettin B for 24 h under a 5% CO_2_ atmosphere at 37 °C, cells were incubated with 10 μM CM-H_2_DCFDA for 30 min. The fluorescent intensity was determined by excitation at 485 nm and emission at 535 nm using a multi-mode microplate reader (FilterMax F5, Molecular Devices, Sunnyvale, CA, USA). Student’s *t*-test was conducted for statistical analysis.

### 3.11. Western Blot Analysis

Western blot analysis was carried out as described previously with a small modification [24]. The suspension (1 × 10^6^ cells/10 mL) of SF295 cells was incubated with 0, 0.04, 0.2 and 1 μM of stellettin B for 24 h under a 5% CO_2_ atmosphere at 37 °C. The cells were harvested and treated with lysis buffer (50 mM Tris-HCl, pH 7.2; 1% NP-40; 0.25% sodium deoxycholate; 150 mM NaCl; 1 mM EDTA; 1 mM PMSF; 1% proteinase inhibitor cocktail) to furnish a cell lysate. Protein assay was carried out by Bio-Rad protein assay kit. After boiling at 95 °C for 5 min in the sample buffer (0.125 M Tris-HCl, pH 6.8; 10% 2-mercaptoethanol; 4% SDS; 10% sucrose; 5% bromophenol blue), equal amounts of protein were subjected to SDS-Polyacrylamide gel electrophoresis (SDS-PAGE) and then transferred to Immobilon-FL PVDF (polyvinylidene fluoride) membrane. After being blocked, the membrane was exposed to anti-p-Akt, anti-Akt, anti-p-ERK, anti-p-p38 or anti-β-actin, and then to Alexa Fluor 680 anti-rabbit IgG secondary antibodies. Signals from the bound labeled-antibodies were detected using the Odyssey Infrared Imaging System (LI-COR Biosciences, Lincoln, NE, USA).

### 3.12. Homogenous Time-Resolved Fluorescence (HTRF) PI3K Assay

The HTRF assay was carried out as described previously [16]. Briefly, the recombinant PI3Kα was incubated with various concentrations of stellettin B, in presence of 10 μM PIP2 in the wells of a 384-well plate at room temperature. The reaction was initiated by addition of 10 μM ATP and was stopped after 30 min of incubation by adding the stop solution containing EDTA and biotin-PIP3. Detection buffer was then added to each well and the resulting mixture was further incubated for 14 h. Signals were read using a multi-mode microplate reader (FilterMax F5, Molecular Devices, Sunnyvale, CA, USA). The PI3K activity of a certain sample was calculated according to the following formula: PI3K activity (% control) = (sample − minus-enzyme control)/(plus-enzyme control − minus-enzyme control) × 100. For the plus-enzyme control, the kinase was incubated with PIP2 and ATP, and for the minus-enzyme control, PIP2 was incubated with ATP only. Representative data from two independent experiments, each carried out in triplicate, were used for plotting. Student’s *t*-test was conducted for statistical analysis.

## 4. Conclusions

Stellettin B, an isomalabaricane-type triterpene isolated from marine sponge *Jaspis stellifera*, exhibited antiproliferative activity on 39 human cancer cell lines. Among the cell lines, stellettin B showed highly potent inhibitory activity on the proliferation of human glioblastoma SF295 cells. More interestingly, stellettin B showed almost no cytotoxic activity on 4 normal cell lines, suggesting the relatively selective cytotoxicity against human cancer cells compared to normal human cell lines. Treatment with stellettin B induced apoptosis, enhanced caspase 3/7 activity, cleavage of PARP, and ROS production. Meanwhile, similar concentrations of stellettin B inhibited the phosophorylation of Akt in SF295 cells, suggesting that inhibition of Akt pathway might be involved in the antiproliferative and apoptosis-inducing effect of stellettin B. However, our biochemical assay result showed that stellettin B did not inhibit PI3K activity, suggesting that the direct target of stellettin might be an upstream signal protein of an Akt pathway other than PI3K. Stellettin B might be a lead compound for discovery of a promising drug candidate for treatment of a part of glioblastomas.
